# A Geometric Morphometric Study of Scapular Ontogeny in Modern Humans

**DOI:** 10.1002/ajpa.70090

**Published:** 2025-07-08

**Authors:** Erica Noble, John Hawks

**Affiliations:** ^1^ Department of Anthropology University of Wisconsin‐Madison Madison Wisconsin USA

**Keywords:** development, hominin evolution, human growth, shoulder

## Abstract

**Objectives:**

This paper quantifies the size and shape change of the human scapula through ontogeny to better understand the human trajectory of growth. While previous work has touched on human scapular ontogeny, analysis using 3D geometric morphometrics focusing on humans alone has not been conducted. This work is important to improve our analyses of the immature hominin fossil record.

**Methods:**

Deidentified CT scans of human nonadults (infancy to adolescence) and adults from The Cancer Imaging Archive were examined in this study. Twenty‐one digital landmarks were placed on the scapula and analyzed using linear regression and geometric morphometrics.

**Results:**

The size of the scapula starts small compared to body size and grows faster than femur head diameter, used as a proxy for body size. Some features that distinguish humans from great ape scapulae also exhibit developmental change in children, notably the angle of the scapular spine. Nonadults have more laterally oriented scapular spines than adults. This suggests that the development of the adult human scapula starts from a shape that is different from other apes and converges slightly during growth, a finding supported by previous work.

**Conclusions:**

These results expand upon our understanding of the development of the human shoulder and our interpretations of juvenile scapulae in the hominin fossil record. Human juveniles, who climb and engage in arboreal behavior more frequently than adults, have a scapula whose morphology is poorly suited to arboreal movement. Whether this is evolutionarily or functionally driven will be explored in further studies using comparative analyses.

## 1 Introduction

The shape of the scapula varies among primate species, and previous studies on the shoulder have confirmed that much of this anatomical variation is related to variation in function (Ashton and Oxnard 1964; Green 2013; Larson 1995; van Beesel 2022; Young 2008). The position of the scapula on the trunk integrates it with muscles of the neck and back, chest, and arm, and its shape reflects the actions and orientations of those muscles, which are frequently used in locomotion. Many previous studies have found that features of the scapula reliably separate the primates into functional groups like quadrupeds, suspensory primates, and brachiators (Inman et al. 1944; Larson 1993; Young 2006). Differences in the scapula between the great apes have also been shown to be diagnostic of locomotor pattern (Larson 1998; Roberts 1974; Shea 1986; Taylor and Slice 2005). Humans are no exception to this pattern: when compared to the scapulae of living great apes, which are superoinferiorly long and more cranially oriented, the human scapula is mediolaterally wide and more laterally oriented. This difference has been ascribed to the difference in shoulder use in humans compared to the other great apes (Larson 2007, 2009; Roach et al. 2013).

While many studies have considered the adult scapula, fewer have looked at scapular morphology in infants, juveniles, and adolescents (referred to from here on as nonadults). The function of the scapula in hominoids must change to some extent across ontogeny as behavior and body size develop, raising the question of how the shape of the scapula in adult hominoids results from development. Several studies have considered the ontogeny of the scapula in great apes and humans (Barros 2014; Green 2013; Green and Alemseged 2012; Young 2006, 2008). Some of these studies have addressed the development in humans, but a missing element has been the detailed consideration of the human developmental trajectory.

One study performed by Green (2013) included humans in an analysis of the change in features of the scapula that are known to vary between functional groups to see if these changes correlated to changes in locomotor pattern. Green's analysis of the human scapula compared to other apes revealed a pattern of shape change that seemed to be distinct. The human juvenile scapular spine and glenoid fossa are more laterally oriented than those of a human adult, although human scapulae at every age stage remain distinct from other apes (Green 2013). These results suggest a surprising contradiction in behavior and form of the juvenile human scapula.

Climbing, especially climbing in trees, is an essential part of human childhood. Frequent climbing assists in the development of balance and motor skills (Căsăneanu et al. 2025), and engaging in tree climbing helps children grow socially and creatively (Gull et al. 2018). Children also climb trees in hunter‐gatherer societies, where they are often tasked with retrieving honey and other high‐energy food like fruit (Kraft et al. 2014). These lines of evidence suggest that climbing is an important part of development, both cognitively and physically, for human children. The presence of scapular features that suggest less capacity for arboreal movement (more laterally oriented spine and glenoid fossa) during a period when humans are most frequently arboreal and using vertical climbing behaviors is unexpected and was the impetus for our present investigation.

Important previous studies (Barros 2014; Green 2013; Green and Alemseged 2012; Taylor 1995; Young 2006, 2008) on scapular ontogeny in hominoids have focused on the scapula in isolation, without additional measurements to track the growth of the rest of the body. While this method of data collection increases the sample size that is possible, mapping the size change of the scapula compared to itself can only reveal so much information. An additional limitation of previous studies was the methodology that only contained linear measurements and indices. Landmark data assessed from 3D imaging modalities enable a more comprehensive picture of shape development than linear measurements or angles alone (Taylor and Slice 2005). These two inclusions (body size data and 3D geometric morphometric analyses) form the basis of our strategy to more fully understand human scapular ontogeny. Here we consider the developmental trajectory of the shape of the human scapula with a sample of nonadults across the span of development to maturation. We examine two questions to compare the human developmental trajectory to previous work on nonhuman hominoids:

### 1.1 Question 1

How much does the relative size of the scapula change through ontogeny?

Human infants are born with proportionally large skulls relative to their body size, a feature that allows for extreme brain growth over the first few years of life (Shonkoff and Phillips 2000). Because of the scapula's position as an insertion point for muscles that hold up the head, scapula growth may be developmentally correlated with skull growth, such that both are relatively larger in young humans. However, many previous studies of scapular ontogeny (Green 2013; Taylor and Slice 2005; Young 2008) do not include direct comparisons to body size in their analyses. We aim to quantify this change in scapular size and determine how the rate of growth of the scapula compares to other skeletal measurements more closely correlated to body size, such as femoral head diameter or rib cage measurements.

Hypothesis 1.1 : *The relative size of the scapula compared to the size of the femoral head and ribcage (as proxies for body size) decreases through ontogeny, as younger humans have larger scapulae relative to their body*.

Hypothesis 1.2 : *The relative dimensions of the scapula will change through ontogeny, with these size changes including nonadults having mediolaterally wider scapulae than adults, following the observation by* Green (2013) *that younger humans are more extreme in their diagnostic scapular traits*.

### 1.2 Question 2

How does the shape of the human scapula change through ontogeny?

With our second research question, we examine specifically the human pattern of development of scapula shape to understand how it relates to growth in body size. As noted above, Green (2013) found that human nonadults follow a counterintuitive pattern of shape change of the scapula, with young infants showing a “hyper‐human” shape that is not seen in the other living apes. We examined how this pattern develops into the adult shape across ontogeny in a cross‐sectional sample of humans from infancy to adulthood.

Hypothesis 2 : *The null hypothesis for this pattern seen in humans is that the shape change is a simple consequence of allometry, with body size determining scapular shape. If this null hypothesis is supported, we expect to see smaller (younger) humans with a scapula shape as described by* Green (2013)*: scapular spines that are less oblique and glenoid fossae that are more laterally oriented than in larger (older) humans. If this is a simple case of allometry, we also expect to see smaller adults with less oblique scapular spines and more laterally oriented glenoid fossae than larger adults*.

## 2 Materials and Methods

### 2.1 Sample

Nonadult data were collected from the Pediatric Chest/Abdomen/Pelvic CT Exams with Expert Organ Contours study, which can be accessed at The Cancer Imaging Archive (Clark et al. 2013) under the collection name Pediatric‐CT‐SEG (Jordan et al. 2022). This study contains CT scans of the torso of human children of various ages from 5 days to 16 years. Individuals are from pediatric cases in various teaching hospitals from Wisconsin and California. Adult data were collected from the Stony Brook University COVID‐19 Positive Cases study on The Cancer Imaging Archive under the collection name COVID‐19‐NY‐SBU (Saltz et al. 2021). Thirty scans of nonadults and 16 scans of adults were collected (Table S1).

### 2.2 Data Collection

**FIGURE 1:**
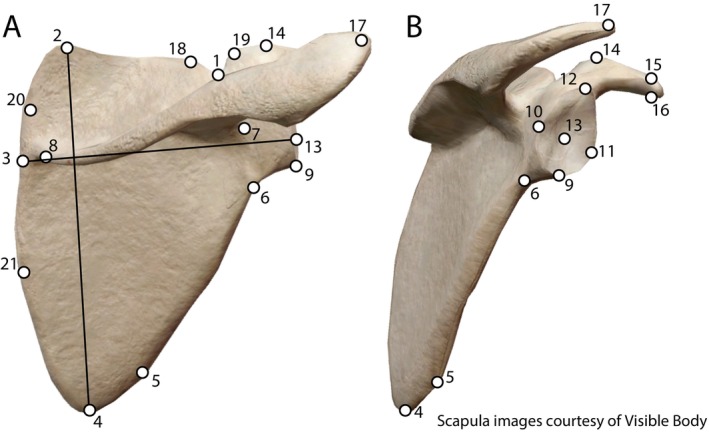
Landmarks and initial measurements on the scapula used in this study from a posterior (A) and lateral (B) view. Landmarks are adapted from Green (2013) and Young (2008) with the exceptions of landmarks 18–21, which were added by the authors. Superoinferior breadth and mediolateral length are represented as lines between landmarks 2 and 4 and landmarks 3 and 13, respectively.

Data were collected on the right scapula, right femur, left innominate, and articulated ribcage and spine for all individuals. Landmarks were placed on 3D mesh files of the relevant bones that were created using the CT scans in the CT visualization software 3D Slicer (Fedorov et al. 2012). Main data were obtained in the form of 21 digital landmarks placed on the scapula (Figure 1A,B; Table 1) following past studies of scapular morphological variation, specifically work on the ontogeny of scapular variation in anthropoids (Green 2013; Taylor 1995; Taylor and Slice 2005; Young 2008). These landmarks were chosen by previous authors for their replicability on both infant and adult scapulae, as some features, such as the acromion and coracoid processes and inferior angle, do not fuse until late in development (see Cunningham et al. 2016). Landmarks corresponding to these features were placed in the best position possible (i.e., placing the landmark for the acromion process on the lateral‐most extension of the scapular spine). Four landmarks were added for this study to increase the resolution of scapula shape when analyzing landmark coordinates: the medial and lateral border of the suprascapular notch (landmarks 18 and 19, respectively), and the most medial aspect of the vertebral border, superior and inferior to where the spine connects to the border (landmarks 20 and 21, respectively).

**TABLE 1:** List of landmarks used for this study with a description of their placement notes.

Landmark	Name	Description	Source
1	Suprascapular notch	The center of the suprascpular notch	Green 2013; Young 2008
2	Superior angle	The superior‐most aspect of the superior angle of the scapula	Green 2013; Young 2008
3	Vertebral margin at long axis of spine	Point of vertebral border at which the long axis of the scapular spine intersects	Green 2013; Young 2008
4	Inferior angle	The inferior‐most aspect of the inferior angle of the scapula	Green 2013; Young 2008
5	Teres major fossa	The lateral‐most expansion point of the lateral border near the inferior angle	Young 2008
6	Infraglenoid tubercle	The lateral‐most expansion point of the lateral border inferior to the glenoid fossa	Green 2013; Young 2008
7	Spinoglenoid notch	Meeting point of scapular spine and scapular blade	Green 2013; Young 2008
8	Medial extent of trapezius	Point where scapular spine meets the scapular blade or thins out to no longer be a spine	Young 2008
9	Inferior glenoid	Inferior‐most aspect of glenoid fossa border	Green 2013; Young 2008
10	Dorsal glenoid	Dorsal‐most aspect of glenoid fossa border	Green 2013; Young 2008
11	Ventral glenoid	Ventral‐most aspect of glenoid fossa border	Green 2013; Young 2008
12	Superior glenoid	Superior‐most aspect of glenoid fossa border	Green 2013; Young 2008
13	Glenoid fossa	Central point of glenoid fossa halfway between landmarks 10 and 11	Young 2008
14	Coracoid prominence	Ventral‐most projection of the medial coracoid	Green 2013; Young 2008
15	Superodistal coracoid	The superior‐most aspect of the distal coracoid process	Young 2008
16	Inferodistal coracoid	The inferior‐most aspect of the distal coracoid process	Young 2008
17	Distal acromion	The distal‐most (lateral‐most) aspect of the acromion	Green 2013; Young 2008
18	Medial suprascapular notch	The medial wall of the suprascapular notch (closest to the rest of the border)	This study
19	Lateral suprascapular notch	The lateral wall of the suprascapular notch (closest to the rest of the border)	This study
20	Medial curve above landmark 3	Point of vertebral border above the scapular spine that is most medial	This study
21	Medial curve below landmark 3	Point of vertebral border below the scapular spine that is most medial	This study

Additional data were collected in the form of linear measurements of the scapula, rib cage, and femur. In accordance with previous work (Green 2013; Shea 1986; Taylor 1995; Young 2008) measurements of the mediolateral length of the scapula and the superoinferior breadth of the scapula were taken in the statistical computing software R (Ihaka and Gentleman 1996; R Core Team 2024), measuring the Euclidean distance between landmarks 3 and 13 and landmarks 2 and 4, respectively (see Figure 1). We also took measurements of the superoinferior length and mediolateral breadth of the rib cage using the markup tool in 3D Slicer. These were calculated by measuring the distance from the spinous processes of T1 to T12 and the distance between the sternal ends of rib 10, respectively (Figure S1A,B). The sternal ends of rib 10 were chosen as the landmarks for calculating mediolateral breadth, as they were easily reproducible using the resolutions available through the CT scans. These measurements were used to create proportions of the scapula and rib cage and quantify the size change of the scapula compared to different areas of the body through ontogeny. Femoral head diameter was also calculated as a proxy for body size (see below).

### 2.3 Creating Proxies

Although we had access to calendar ages in this dataset, future planned studies will use data comprising individuals with no known age (such as museum hominoids and fossil specimens). This dataset provided an opportunity to develop a categorical assessment of age and test its efficacy using individuals with known ages. Four phases of development were determined based on the fusion of the three bones of the innominate (see Verbruggen and Nowlan 2017 for a review). The fusion of these bones (pubis, ilium, and ischium) occurs in a set order throughout childhood (Figure S1D). Phase One, determined to be before any fusion begins with three separate bones, occurs in children who are 5 years old or younger. Phase Two, in which the pubis and ischium are close to connecting, occurs in children who are around 5 or 6 years old. Phase Three, in which the pubis and ischium have completely fused and the acetabulum is beginning to fuse around the ilium and ischium, occurs in children who are between 9 and 11 years old. Phase Four represents a completely fused innominate, which is in place by mid‐puberty (12–13 years old for females, 14–15 years old for males).

We found that the developmental phases protocol successfully sorted infants and adolescents (Phases One and Four). However, there was substantial overlap in actual vs. expected phase for juveniles (Phases Two and Three) given the age ranges of innominate fusion that we used for estimation of developmental phase (Table S2). This protocol consistently overestimated the development of individuals in Phase Two, and occasionally did the same for individuals in Phase Three. The provenance of the nonadult dataset may be affecting this; data were obtained from pediatric cases at Children's Hospitals. The individuals in this dataset therefore may not represent accurate growth rates of the general population.

To compare the growth of the scapula to the growth of the body, femoral head diameter was used as a proxy for body size. A previous study (Wegener et al. 2017) has shown that the increase in size of the maximum diameter of the femoral epiphysis parallels growth charts throughout development, making the femoral head diameter a useful way to estimate body size when height and weight data are unavailable. Because younger individuals do not have ossified epiphyses, the diameter was taken at the base of the femoral head, which is present in all phases. The diameter was taken using the markup tool in 3D Slicer. For consistency, an anteroposterior diameter was calculated (Figure S1C).

### 2.4 Data Analysis

Data analyses were performed in R and MorphoJ (Klingenberg 2011). Linear regression analyses were performed on the linear measurements of the scapula, rib cage, and femur to determine if significant changes in scapula size compared to other aspects of the body occurred through development. The mediolateral dimensions of the scapula and rib cage were compared to each other, as were the superoinferior dimensions. Linear regression was also performed on scapula size and rib cage proportions compared to femoral head diameter to see if either element had a different pattern of growth than body size.

### 2.5 Geometric Morphometrics

A generalized Procrustes analysis was run on the landmark data using the R package Morpho (Schlager 2017), which removed variation in the dataset due to translation, rotation, and size differences between landmark configurations and produced coordinates that varied in shape only. These Procrustes coordinates were also used to scale the data to a consistent standard.

To assess the influence of scapular size on shape, we performed a multivariate regression of the Procrustes shape variables onto the natural log of centroid size in MorphoJ. The natural log of centroid size was used in lieu of actual centroid size as is the protocol for ontogenetic studies, where the largest changes in size are restricted to a small range of the data (i.e., very young individuals) (Fernandez Blanco et al. 2018; Mitteroecker et al. 2004; Mitteroecker and Bookstein 2008). This analysis included a permutation test with 10,000 rounds of permutation, which determined independence between shape and scapular size (*p* < 0.0001). A similar test was performed on shape variables and proxies of body size (femoral head diameter, rib cage length, and breadth).

Principal component analyses (PCAs) were run on the shape coordinates created by the Procrustes analysis using Morpho. We performed a shape space PCA as well as a form space PCA to capture variation in shape that is linked to size. Shape space PCA is the analysis of the scaled Procrustes shape coordinates, while a form space analysis includes the natural log of centroid size along with the shape coordinates. The advantage of a form space PCA is that it allows us to capture all variation correlated with size in the first principal component (see Mitteroecker et al. 2004 for an explanation of form space analysis).

Shape change across component axes was visualized by warping the mean shape of the dataset calculated by the Procrustes analysis by two standard deviations in the positive and negative directions of the axis using the eigenvectors and eigenvalues of that component. The resulting landmark coordinates were used to create a wireframe representation of scapular shape.

### 2.6 Identifying Developmentally Relevant Features

**FIGURE 2:**
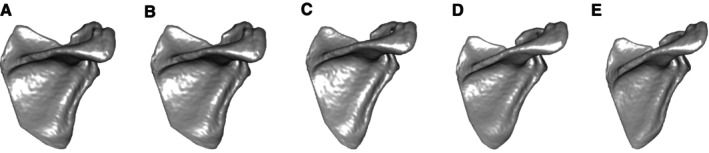
Mesh representations of the mean shape of the data warped to the mean shape of each phase. Phases are arranged as follows: 1 (A), 2 (B), 3 (C), 4 (D) and Adult (E).

**FIGURE 3:**
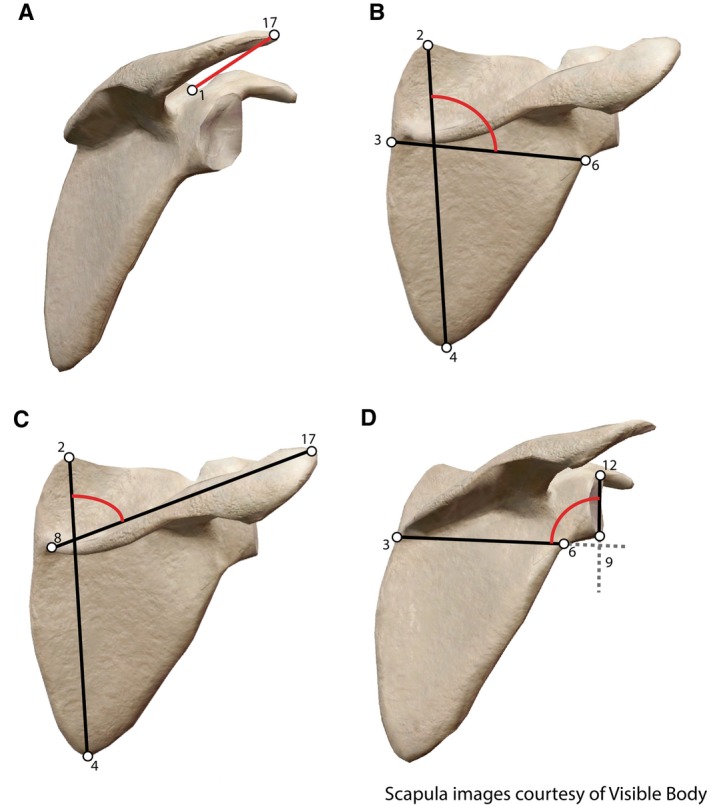
Features of the scapula that change with size (and thus age), referred to as “diagnostic features” for their ability to distinguish between individuals of varying sizes. (A) Acromial distance (the distance from the acromion to the suprascapular notch). (B) Blade angle (angle of the mediolateral blade axis on the superoinferior blade axis). (C) Spine/SI blade angle (angle of the scapular spine on the superoinferior axis of the scapular blade). (D) Glenoid/ML blade angle (angle of the glenoid fossa on the mediolateral axis of the scapular blade).

Average shape coordinates were calculated for each developmental phase. These coordinates were used to warp the mesh of the mean shape of the dataset using the tps3D function within the R package Morpho to create a representation of scapular shape at that developmental phase (Figure 2A–E). The mesh figures were visually analyzed along with the eigenvectors of each landmark coordinate for the first component of the form space PCA to determine the changes in features that are most associated with development. These were translated into biologically significant features that were measured between the landmarks of interest, all of which are presented in Figure 3.

The linear measurement that was identified using these methods was the distance between the acromion process and the suprascapular notch (as a stand‐in for the location of the scapular blade). This length was calculated by measuring the Euclidean distance between landmarks 1 and 17 (Figure 3A). The angles that were identified were of the scapular dimensions, scapular spine, and glenoid fossa. These angles were calculated using the dot‐product formula for finding angles between vectors in 3D space. The angle of the mediolateral blade on the superoinferior blade was calculated using vectors from lines between landmarks 3 and 6 and landmarks 2 and 4 (Figure 3B). The angle of the scapular spine on the scapular blade was calculated using landmarks 8 and 17 and landmarks 2 and 4 (Figure 3C). The angle of the glenoid fossa was calculated using landmarks 3 and 6 and landmarks 9 and 12 (Figure 3D). These measurements were plotted against each other and underwent linear regression analysis to determine which combination produced the best separation of developmental stages for use in future studies.

## 3 Results

### 3.1 Hypothesis 1.1

**FIGURE 4:**
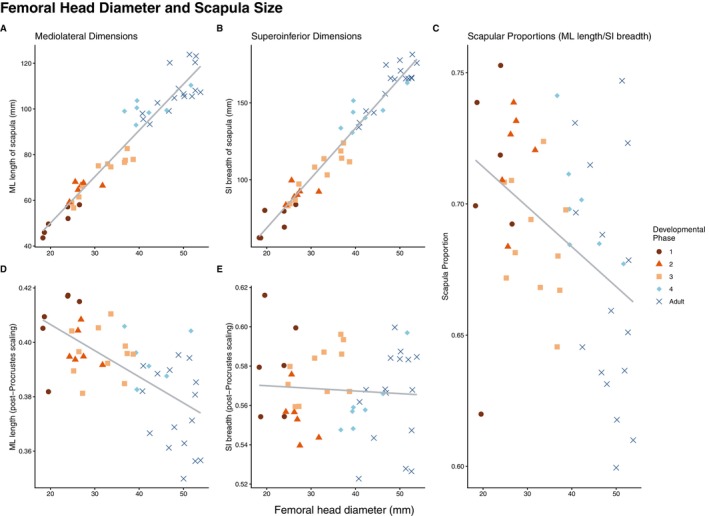
Linear measurements of the scapula compared to femoral head diameter. All x‐axes are femoral head diameter. (A) Measurements of mediolateral length. (B) Measurements of superoinferior breadth. (C) Mediolateral length divided by superoinferior breadth. (D) Measurements of mediolateral length after individuals have been scaled to a mean shape via Procrustes analysis. (E) Measurements of superoinferior breadth after individuals have been scaled to a mean shape via Procrustes analysis. Note that once scaled, the linear measurements become less correlated with body size, with the relationship between mediolateral length and femoral head diameter reversing.

Measurements of scapular length (from landmark 3 to landmark 13) and scapular breadth (from landmark 2 to landmark 4) correlated well with femoral head diameter, as is expected in a sample with a large range in body size (*R* ^2^ > 0.9, Figure 4A,B). Surprisingly, scapular dimensions increased at a faster rate than femoral head diameter did (linear regression slope = 2 for mediolateral measurements [Figure 4A], 3.2 for superoinferior measurements [Figure 4B]). This suggests that the scapula is growing at a faster rate than body size, the opposite of what would be expected if humans are born with relatively large scapulae.

Post‐Procrustes scaling weakens this close relationship between linear measurements of the scapula and body size. Mediolateral length measurements maintain some correlation with femoral head diameter (*R* ^2^ = 0.4, Figure 4D), while superoinferior breadth had no correlation (Figure 4E). Comparing the measurements of the scapula to measurements of the rib cage produces similar results. Direct measurements have strong relationships to one another, although superoinferior measurements (*R* ^2^ = 0.93, Figure S2B) are more tightly correlated to each other compared to mediolateral measurements (*R* ^2^ = 0.76, Figure S2A). The measurements of the scapula increase at a slower rate than the measurements of the rib cage for both mediolateral (linear regression slope = 0.38, Figure S2A) and superoinferior (linear regression slope = 0.65, Figure S2B) dimensions. Compared to the relationships found between the scapula and body size, this suggests that the scapula is growing at a slower rate than the rib cage and that babies are born with relatively large scapulae compared to their rib cage, but small scapulae compared to our proxy for body size.

No strong relationship is present between superoinferior measurements of the scapula and rib cage after the landmarks are scaled to the Procrustes mean (Figure S2E), and proportions of the scapula do not correlate to proportions of the rib cage (Figure S2C). Only scapular mediolateral length compared to rib cage mediolateral breadth produces a correlation once scapula size is standardized (*R* ^2^ = 0.41, Figure S2D).

Hypothesis 1.1 is not supported by our results. The scapula appears to be small at birth compared to body size, as the mediolateral and superoinferior dimensions of the scapula increase at a faster rate through ontogeny than the femoral head diameter (Figure 4A,B), which is not what was expected. However, the scapula is large at birth compared to the rib cage, as its mediolateral and superoinferior dimensions increase at a slower rate than those dimensions of the rib cage (Figure S2A,B). These results suggest that compared to body size, both the scapula and the rib cage are relatively small at birth, with the rib cage being relatively smaller than the scapula. This relationship is supported by a comparison of femoral head diameter and rib cage dimensions directly (Figure S3). Both mediolateral (Figure S3A) and superoinferior (Figure S3B) measurements of the rib cage increase at a much faster rate than the femoral head diameter (linear regression slope = 4.3 and 4.8, respectively).

### 3.2 Hypothesis 1.2

**FIGURE 5:**
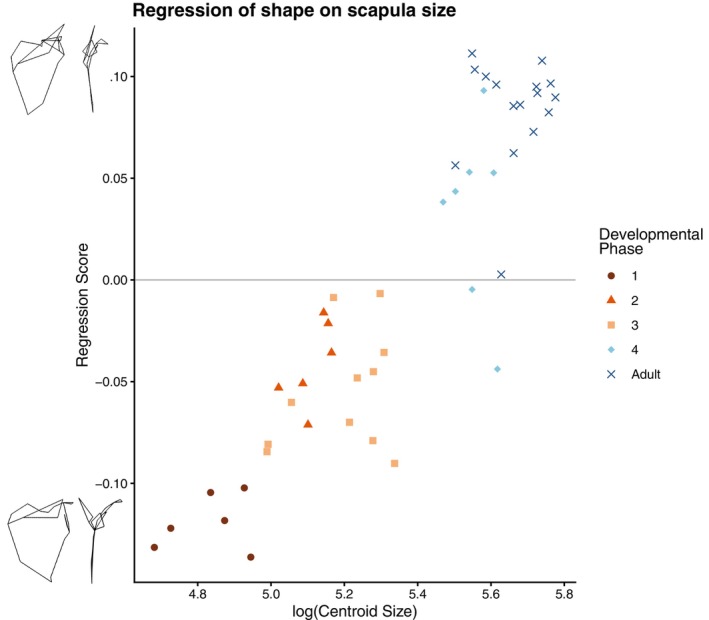
A visual representation of the multivariate regression of Procrustes shape coordinates on the natural log of centroid size. Warps of regression score extremes were obtained using the predicted shape coordinates produced by the regression in MorphoJ. Note the difference in shape between individuals with positive vs. negative regression scores and their correlation to centroid size and developmental phase.

To properly assess the relationship between scapular shape and size, we regressed the shape coordinates onto measures of size including the natural log of centroid size and femoral head diameter (Table 2). We found that log(centroid size) was the best predictor for scapular shape, explaining around 30% of the shape variation (Table 2). Removing centroid size and only using measures of body size outside of the scapula, femoral head diameter predicted around 25% of the shape variation (Table S3). These regressions capture the same type of shape change, with regression scores being highly correlated between individuals (*r* = 0.999). The variation in shape explained by size includes a change in mediolateral length, with smaller individuals having longer scapulae from the vertebral border to the glenoid fossa (Figure 5). Visualizing the variation of shape predicted by size shows a strong linear relationship (*R* ^2^ = 0.81), where smaller individuals have lower regression scores and vice versa (Figure 5).

**TABLE 2:** Results of the multivariate regression of Procrustes shape coordinates on the natural log of centroid size and femoral head diameter. Note the relatively small *R* ^2^ for the femoral head diameter, meaning that it does not explain much more variation not explained by centroid size.

	Df	SS	MS	Rsq	F	Z	Pr (> F)
log(centroid size)	1	0.238	0.238	0.298	19.421	4.366	1E‐04
Femoral head diameter	1	0.035	0.035	0.044	2.851	2.180	0.015
Residuals	43	0.527	0.012	0.659	—	—	—
Total	45	0.7997	—	—	—	—	—

This change in shape is also seen in the analyses of linear measurements. When plotting scapular proportions (mediolateral length divided by superoinferior breadth) against femoral head diameter, a slight relationship (*R* ^2^ = 0.18) is observed wherein younger individuals have proportionally wider scapulae (Figure 4C).

Hypothesis 1.2 is supported by our data. The results of the multivariate regressions of shape variables against size covariates demonstrate a change in shape from relatively mediolaterally long to mediolaterally short scapulae as size (and in an ontogenetic sample like this one, also age) increases. These results follow the general idea that nonadult human scapulae are wider (and thus further down on the “human” scale of diagnostic traits) than adults, as we expected given observations by Green (2013). However, because this is an ontogenetic dataset comprising a wide range of ages and sizes, these results do not confirm whether this variation is allometric or ontogenetic in nature. This will be clarified using methods in Question 2.

### 3.3 Hypothesis 2

**FIGURE 6:**
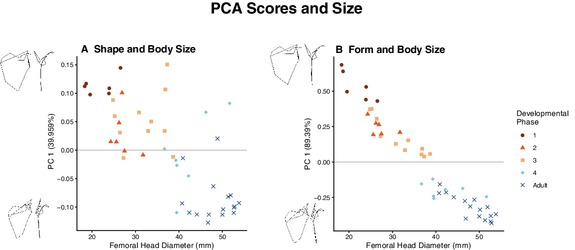
The first component scores of the shape space (A) and form space (B) Principal Components Analyses compared to femoral head diameter. Warps of component score extremes were obtained using the eigenvectors and eigenvalues of the component and warped to 2 standard deviations from the mean.

**FIGURE 7:**
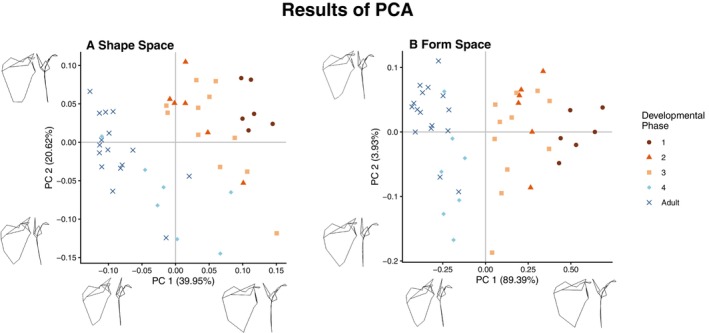
The first and second component scores of the shape space (A) and form space (B) PCAs. Warps of component score extremes were obtained using the eigenvectors and eigenvalues of the component and warped to two standard deviations from the mean. Both analyses capture similar shape change in the first component, as evidenced by the line plot warps.

Our initial PCA of shape coordinates identified variation in shape that correlated to changes in body size (Figure 6A) such that smaller (and younger) individuals had higher PC 1 scores. The first two components captured about 60% of the variation in the sample (Figure 7A), although there was overlap in the distribution of adults and nonadults within the PCA space.

We also performed a form space PCA by including log(centroid size) as a variable along with the shape coordinates. The explicit inclusion of size in the analysis linked more shape variation with change in size, capturing 89% of the variation in the sample in the first component (Figure 7B). This analysis also clearly separated individuals with fully fused innominate bones (adults and members of developmental phase 4) from individuals with unfused or partially fused innominate bones (members of developmental phases 1–3) in the first component, with the former group having negative scores and the latter having positive scores. Because of this increased separation of age groups compared to the shape space analysis, further analyses were performed on the results of the form space PCA.

The relationship between PC 1 scores and size from the shape space analysis was retained in the form space analysis (Figure 6B). The results of this comparison show that even adolescents that are very large in body size have relatively higher PC 1 scores compared to adults of similar or even smaller size. This suggests that the shape changes represented in this first form component are ontogenetically and not simply allometrically based.

Mesh representations of the average shape of each developmental phase, line plots of shape extremes, and eigenvectors of each landmark were used to identify the largest changes in features captured by the first form component. The line plots show that individuals with more positive PC1 scores (smaller/younger individuals) have relatively wider scapulae with scapular spines that are less oblique than larger/older individuals (Figure 7B). The lateral view of the line plots shows that the acromion process also shifts to lie flatter against the blade of the scapula by adulthood, instead of being almost perpendicular as in the first developmental phase.

An analysis of the eigenvectors of form component 1 for each landmark coordinate identified landmarks 2, 3, 4, 9, 14, and 20 as being the most strongly weighted in terms of change across the component axis. Additional landmarks that changed along the axis were landmarks 6, 7, 8, 12, and 19. Using these landmarks and a visual analysis of the form PC1 lineplots, we determined the most significant features that changed with size (as a proxy for age) to be the distance from the acromion process to the suprascapular notch (referred to as acromial distance from here on), the angle between the superoinferior and mediolateral axes of the scapular blade (blade angle) the angle between the scapular spine and the superoinferior axis of the scapula (spine/SI blade angle), and the angle between the glenoid fossa and the mediolateral axis of the scapular blade (glenoid/ML blade angle).

**FIGURE 8:**
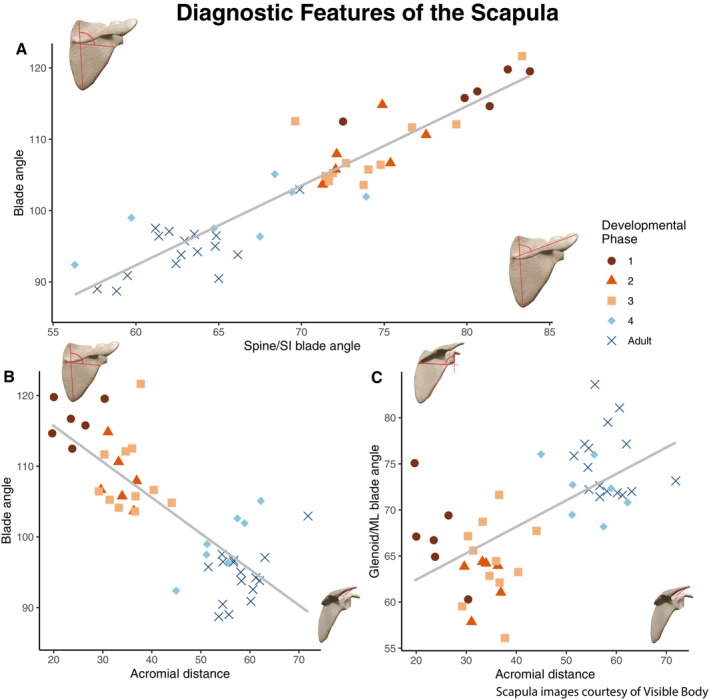
Key features that change through human scapular ontogeny compared against one another. (A) Blade angle (angle of the ML axis on the SI axis of the scapula) and spine/SI blade angle (angle of the scapular spine on the SI axis of the scapula). (B) Blade angle and acromial distance (distance from acromion to suprascapular notch). (C) Glenoid/ML blade angle (angle of the glenoid fossa on the ML axis of the scapula) and acromial distance).

We plotted these features against our measure of body size (Figure S4A–D) and each other (Figure 8A–C). We found that the blade angle and acromial distance were able to visually separate developmental phases best (Figure 8B, *R* ^2^ = 0.66), while the spine/SI blade angle and blade angle correlated best with some visual overlap in developmental phases (Figure 8A, *R* ^2^ = 0.89). These analyses show that (larger) adult and adolescent individuals have a larger acromial‐blade distance with a smaller angle formed between the scapular spine and superoinferior axis, as well as between the superoinferior and mediolateral axes of the scapula. Younger (and smaller) individuals have a smaller acromial‐blade distance with a larger angle between the spine and superoinferior axis, as well as the superoinferior/mediolateral axes. These results parallel the pattern identified by Green (2013) who found that human infants and juveniles had more obliquely angled scapular spines compared to adults.

Glenoid fossa angles were not in line with previous analyses by Green. Smaller individuals generally had smaller angles compared to larger individuals (Figure S4D). This suggests that the glenoid fossa is more cranially oriented in smaller (younger) humans and moves to be oriented closer to a right angle compared to the mediolateral axis of the blade (more laterally oriented) as we grow. The difference between our results and those of Green (2013) may be due to differences in methods of calculating angles. Green used the angle between the glenoid and ventral bar, while we used the angle on the mediolateral blade of the scapula as we did not landmark the ventral bar. Future work will rectify this issue, as visual analysis of the line plots does suggest a shift from more laterally to more cranially oriented glenoid fossae as humans move through ontogeny.

The principal component analyses captured changes to the morphology of the scapula that are correlated with development. In PC 1 of both shape and form analyses, the angle of the scapular spine and glenoid fossa shift from being more laterally oriented in negative scores to being more obliquely oriented in positive scores. This component groups individuals by developmental phase, suggesting that these features are relevant for the trajectory of human scapular development. The line plots created from PC scores also demonstrate clear morphological differences between the youngest and oldest individuals, with differences in mediolateral length, superoinferior breadth, and spine and glenoid angle. These results underscore that developmentally important shape changes occur in the scapula during ontogeny and show that the smallest adult still has an oblique scapular spine compared to large nonadults. This suggests that the pattern found by Green (2013) and this paper is not due to body size alone and has ontogenetic significance.

Identifying these developmentally important features allows us to measure the same features on individuals outside of the original dataset as a comparative sample, which forms the basis of future studies. These results support Hypothesis 2 by grouping all adults together and distinct from all nonadults, suggesting that extreme spine and glenoid angles are not a byproduct of small scapulae and that this pattern is driven by something other than simple allometry.

## 4 Discussion

### 4.1 Scapula Size Through Ontogeny

Our results show that growth and development of the human scapula is not a straightforward consequence of body size growth and development. The size of the scapula compared to the ribcage is large, and small compared to the rest of the body. This may be understood by examining the constraints on the fetal shoulder. The shoulder can become stuck during birth, resulting in potential trauma to the mother and fetus, a condition called shoulder dystocia (Gottlieb and Galan 2007). The shoulder may have been selected to be smaller at birth and then “catch up” to body size during postnatal growth. This suggests that the size of the scapula may not be regulated by the size of the skull, but by obstetric constraints.

### 4.2 Scapula Shape Through Ontogeny

The development of scapular shape in the great apes and other primates remains relatively static, with distinctive traits present at birth and little age‐related change (Green 2013; Young 2008). The strongest phylogenetic signals within families are present in infancy, followed by convergence in shape between primates with similar locomotor functionality as growth progresses (Young 2008). However, these scapular shape changes that can be linked to a response to a change in locomotor behavior are not substantial enough to confuse one species with one another (Green 2013). Humans present a distinctive trajectory of scapular shape change, with a change in scapular spine orientation that is unlike the other primates. The human trajectory is unique and counterintuitive in that it implies that infants are “hyper‐human” in their scapular shapes and adults are more apelike in contrast. Our analysis shows that this pattern of shape change is not consistent with allometry resulting from size growth across ontogeny. This implies that the shape change trajectory may have a functional interpretation beyond simple growth of the body, potentially due to the way that nonadults use their shoulders compared to adults.

### 4.3 Interpretations of the Fossil Record

The results of this study provide an important framework to reconsider hominin fossil scapulae, as some of the most complete scapulae (DIK‐1‐1, ATD6‐118 and 116, and KNM‐WT 15000) are of nonadults (Alemseged et al. 2006; García‐Martínez et al. 2021; Walker and Leakey 1993). By better understanding the human trajectory of scapular morphology through ontogeny, we can recontextualize juvenile fossils that are following the human pattern of development. The case of the KNM‐WT 15000 (“Nariokotome boy”) partial skeleton assigned to *Homo erectus* is especially of renewed interest with the findings from this research. Age estimates for the Nariokotome boy vary depending on the growth model used (Dean and Smith 2009), making it difficult to assess the growth pattern of *Homo erectus*. Estimates range from early adolescent at the oldest and prepubescent at the youngest, placing him around Phase Three of this study. Analyses of the scapular spine of KNM‐WT 15000 have suggested that it is much more horizontal in orientation than earlier hominins, and even more horizontal than a population of modern European Americans (Larson 2007). This hyper horizontal scapular spine is in line with what is expected of a human adolescent—more extreme features than their adult counterparts. This could strengthen the argument that Nariokotome is following a human‐like growth trajectory, and his chronological age is closer to his skeletal age of around 13 years old, rather than his dental age of 10 years (Dean and Smith 2009).

### 4.4 Sample Limitations

The nature of the sample used in this study presents natural limitations that must be addressed. Individuals had identifying information removed from the main database that they were accessed from, meaning that demographic information such as sex was not present. The exclusion of sex data may obscure sources of variation, as sexual dimorphism trends through ontogeny have been recorded in regions of the skeleton that are linked to the scapula, including the thorax (García‐Martínez et al. 2020). However, this sexual dimorphism is not present until early puberty, and other studies on the ontogeny of the thorax do not identify strong signals of sexual dimorphism in human infants or juveniles (Bastir et al. 2013; García‐González et al. 2023). These studies suggest that there may be some variation between sexes during adolescence, but that males and females are not easily differentiated during early childhood. Future studies with larger sample sizes may illuminate sex as another source of variation in the ontogenetic path of the scapula, but if its effects follow those of related elements, it may account for only a small amount of variation, especially in early ontogeny.

Additionally, the extremely small sample sizes of the developmental phases made within‐group analyses impossible. All analyses presented examine the dataset as a whole and look at relationships across the range of ontogeny. Future studies will increase the sample size within each developmental phase to assess variation between different stages of development.

### 4.5 Future Studies

The results of the test of developmental phase efficacy suggest that the protocol for estimating development should be reworked to include different growth standards. However, it represents a solid starting point for estimating age without dental eruption, a challenge that makes analyzing fossil hominin ages difficult. Additionally, the first few years of life are very developmentally significant, and they are currently all grouped together in Phase One. This study may be obscuring important stages of scapular shape development by treating infancy and young childhood as one group. Future studies of this dataset will use a different method of developmental phase estimation using the spine to increase resolution in the first few years of life. A review of the ontogeny of the spine (Martelli 2019) provides multiple avenues for estimating age, including the length of each region of the spine, the development of cervical and lumbar lordosis, and the fusion of arch elements in different parts of each spinal region. This holistic method of age estimation will allow for more precise and accurate estimations than could be obtained from using innominate fusion and includes developmental periods from as young as birth up to 18 years and beyond. Martelli (2019) also describes hominoid spinal ontogeny, including arch fusion, which will allow for consistent age estimation in comparative ape samples used in future studies.

The results from this study are weakened without a comparative sample to put this ontogenetic pattern into context. Now that we have a clear picture of the developmentally important features in humans, we can compare humans to other hominoids directly. A comparison with great ape adult and nonadult scapular specimens in future studies will allow us to determine the degree to which this pattern of scapular growth is unique to humans, or if nonhuman great apes have similar patterns or trajectories. This comparative analysis will allow us to determine the degree to which scapular ontogeny in modern humans is functionally driven.

The ontogenetic comparisons considered in this study suggest that nonadults may be an important element of understanding the evolution of scapular morphology. Nonadults are different in body size from adults, and their skeletal structure and body plan may place different requirements on the scapula. The development of the human locomotor pattern, including both the development of climbing and of bipedal movement, exerts a unique set of functional demands on the shoulder in comparison with other primates who move quadrupedally. A future study of human and orangutan scapulae provides an interesting potential morphological comparison, as the orangutan locomotor pattern also includes bipedal postures and climbing behavior (Manduell et al. 2011).

## 5 Conclusion

The results of this study suggest that the scapula undergoes a specific change in shape that is separate from any biomechanical consequences of the proportions and size of the juvenile body compared to the adult body. These results also visualize and quantify this change, which is primarily constrained to the orientation of the scapular spine and the width of the scapula. Previous studies of interspecific variation in the scapula have shown that humans have a mediolaterally wide scapula with a horizontal scapular spine compared to other African apes (Ashton and Oxnard 1964; Larson 2009). The results of this study suggest that these features that are considered indicators of human morphology are more pronounced in infants and juveniles compared to adolescents and adults. It does not seem to be a pattern that is replicated in small adults compared to large adults and cannot be explained by size differences alone, suggesting this shape trajectory is developmentally relevant. Future studies will provide comparative samples and analyses to determine the mechanisms underlying this trajectory and solidify our knowledge of modern human scapular ontogeny compared to the other apes.

## Author Contributions

**Erica Noble:** conceptualization (equal), data curation (lead), funding acquisition (supporting), methodology (lead), visualization (lead), writing – original draft (lead). **John Hawks:** conceptualization (equal), funding acquisition (lead), methodology (supporting), project administration (lead), resources (lead), supervision (lead), writing – review and editing (lead).

## Ethics Statement

Human data used in this study were collected from CT scans available on The Cancer Imaging Archive and did not require permission to access. The University of Wisconsin‐Madison IRB reviewed the project and determined that the proposed activity is not research involving human subjects as defined by DHHS and FDA regulations.

## Conflicts of Interest

The authors declare no conflicts of interest.

## Supporting information


**Figure S1.** Additional measurements and data used in this study. (A) Mediolateral breadth of the rib cage, measured between the sternal ends of rib 10. (B) Superoinferior length of the rib cage, measured between the spinous processes of T1 and T12. (C) Anteroposterior diameter of the femoral head. Diameter taken from the base of the femoral head, as this part of the femur was present in all phases. (D) Representatives of the different stages of innominate fusion that were used to assign individuals to a developmental phase. From left to right: Phase 1, Phase 2, Phase 3, Phase 4.

**Figure S2.** Linear measurements of the rib cage compared to measurements of the scapula. (A) Rib cage mediolateral breadth and scapula mediolateral length. (B) Rib cage superoinferior length and scapula superoinferior breadth. (C) Rib cage proportions and scapula proportions. (D) Rib cage mediolateral breadth and Procrustes‐scaled scapula mediolateral length. (E) Rib cage superoinferior length and Procrustes‐scaled scapula superoinferior breadth. Because a GPA was not performed on the rib cage dimensions, all measurements of the rib cage are direct/unscaled.

**Figure S3.** Dimensions of the rib cage compared to femoral head diameter. (A) Superoinferior length. (B) Mediolateral breadth.

**Figure S4.** Diagnostic features of the scapula compared to femoral head diameter to show their ability to differentiate between sizes (and thus ages). (A) Acromial distance. (B) Spine/SI blade angle. (C) Blade angle. (D) Glenoid/ML blade angle.

**Table S1.** Sample size for each developmental phase.

**Table S2.** Distribution of ages of individuals in each developmental phase. Groups of ages were determined using the age ranges of each state of innominate fusion as identified in Verbruggen and Nowlan (2017). Italic numbers represent individuals who were correctly assigned to the developmental phase that corresponds to their age. Note that most misidentification occurs via assigning individuals to a phase with age ranges older than they are. Classification accuracy: 73.91%.

**Table S3.** Results of a multivariate regression of Procrustes shape coordinates on measures of body size outside of the scapula. Rib cage dimensions do little to improve the model’s ability to explain variation outside of femoral head diameter alone.

## Data Availability

The CT scan data that support the findings of this study are openly available in The Cancer Imaging Archive at https://doi.org/10.7937/TCIA.BBAG‐2923 and https://doi.org/10.7937/TCIA.X0H0‐1706, free to access. Landmarks and other supporting data can be accessed on FigShare at https://figshare.com/projects/A_geometric_morphometric_study_of_scapular_ontogeny_in_modern_humans/253076.
