# Supplementary material for: A Geometric Morphometric Study of Scapular Ontogeny in Modern Humans
Source: Am J Biol Anthropol. 2025 Jul 8;187(3):e70090. doi: 10.1002/ajpa.70090 (PMC12236271; doi:10.1002/ajpa.70090)
Supplement: Supplementary file 5 — Table S1. Sample size for each developmental phase. [file AJPA-187-e70090-s006.docx]

| Developmental Phase | N |
| --- | --- |
| 1 | 6 |
| 2 | 6 |
| 3 | 11 |
| 4 | 7 |
| Adult | 16 |
| Total | 46 |

Table S1: Sample size for each developmental phase.
