# Supplementary material for: A Geometric Morphometric Study of Scapular Ontogeny in Modern Humans
Source: Am J Biol Anthropol. 2025 Jul 8;187(3):e70090. doi: 10.1002/ajpa.70090 (PMC12236271; doi:10.1002/ajpa.70090)
Supplement: Supplementary file 6 — Table S2. Distribution of ages of individuals in each developmental phase. Groups of ages were determined using the age ranges of each state of innominate fusion as identified in Verbruggen and Nowlan (2017). Italic numbers represent individuals who were correctly assigned to the developmental phase that corresponds to their age. Note that most misidentification occurs via assigning individuals to a phase with age ranges older than they are. Classification accuracy: 73.91%. [file AJPA-187-e70090-s002.docx]

| Actual Age Range | Assigned Phase 1 (0-5 years) | Assigned Phase 2  (6-8 years) | Assigned Phase 3  (9-11 years) | Assigned Phase 4  (12-18 years) | Assigned Adult  (>18 years) | Total |
| --- | --- | --- | --- | --- | --- | --- |
| 0-5 years | *6* | 5 | 3 | 0 | 0 | **14** |
| 6-8 years | 0 | *1* | 3 | 0 | 0 | **4** |
| 9-11 years | 0 | 0 | *4* | 0 | 0 | **4** |
| 12-18 years | 0 | 0 | 1 | *7* | 0 | **8** |
| >18 years | 0 | 0 | 0 | 0 | *16* | **16** |
| Total | **6** | **6** | **11** | **7** | **16** | **46** |

Table S2: Distribution of ages of individuals in each developmental phase. Groups of ages were determined using the age ranges of each state of innominate fusion as identified in Verbruggen & Nowlan (2017). Italic numbers represent individuals who were correctly assigned to the developmental phase that corresponds to their age. Note that most misidentification occurs via assigning individuals to a phase with age ranges older than they are. Classification accuracy: 73.91%.
