# Supplementary material for: A Geometric Morphometric Study of Scapular Ontogeny in Modern Humans
Source: Am J Biol Anthropol. 2025 Jul 8;187(3):e70090. doi: 10.1002/ajpa.70090 (PMC12236271; doi:10.1002/ajpa.70090)
Supplement: Supplementary file 7 — Table S3. Results of a multivariate regression of Procrustes shape coordinates on measures of body size outside of the scapula. Rib cage dimensions do little to improve the model’s ability to explain variation outside of femoral head diameter alone. [file AJPA-187-e70090-s005.docx]

|  | Df | SS | MS | Rsq | F | Z | Pr(>F) |
| --- | --- | --- | --- | --- | --- | --- | --- |
| Femoral head diameter | 1 | 0.201 | 0.201 | 0.251 | 15.075 | 4.063 | 1E-04 |
| SI rib cage length | 1 | 0.028 | 0.0278 | 0.035 | 2.081 | 1.651 | 0.053 |
| ML rib cage breadth | 1 | 0.011 | 0.011 | 0.014 | 0.856 | -0.028 | 0.508 |
| Residuals | 42 | 0.560 | 0.013 | 0.699 |  |  |  |
| Total | 45 | 0.7997 |  |  |  |  |  |

Table S3: Results of a multivariate regression of Procrustes shape coordinates on measures of body size outside of the scapula. Rib cage dimensions do little to improve the model’s ability to explain variation outside of femoral head diameter alone.
